# Safety of Compounded Medications

**DOI:** 10.7759/cureus.78541

**Published:** 2025-02-05

**Authors:** Rohini Garg, Harmit Singh

**Affiliations:** 1 Department of Internal Medicine, CHI Health Mercy Hospital, Council Bluffs, USA; 2 Department of Psychiatry, CHI Health Mercy Hospital, Council Bluffs, USA

**Keywords:** compounded medications, compounded medications adverse effects, compounded medicines, compounded versus fda approved medications, safety of compounding

## Abstract

Compounded medications are medications that are prepared by compounding pharmacies to meet individual patients' needs that cannot be met by FDA-approved brand name or generic medications. This may be the case when a patient needs a different dose or route of administration or if a patient is allergic to a certain ingredient in an FDA-approved medication. While compounded medications help to fill a necessary gap, they can have significant safety issues. Due to lesser regulatory oversight, patient safety may be compromised due to contamination or inaccurate potency of compounded medications. Compounded medications should be prescribed only when an FDA-approved medication cannot be used, and the benefits outweigh the risks. Enforcement of adequate oversight by regulatory agencies and maintenance of strict safety precautions at the compounding facilities are needed to ensure patient safety.

## Editorial

Definition of compounded medications and the difference between FDA-approved medications and compounded medications

Compounding is the process of combining, mixing, or altering ingredients of a drug in order to prepare a medication customized for a specific patient [1]. While compounding can provide medications to patients whose needs are not otherwise met with FDA-approved drugs, they may pose significant risks [2,3]. In contrast to the FDA-approved brand name and generic medications, compounded medications have lesser regulatory oversight [2,3]. FDA-approved brand name and generic medications undergo comprehensive testing and a rigorous approval process to ensure efficacy and safety for large-scale use. They are manufactured under Good Manufacturing Practice (GMP) regulations, which are established by federal regulation [4]. In contrast to FDA-approved medications, compounded medications are prepared by individual pharmacies that operate under the state board of pharmacy and are exempt from FDA regulations and scrutiny [2]. Compounded drugs are not assessed by the FDA for quality, safety, or efficacy and, hence, can pose health risks to patients.

Uses and risks of compounded medications

Medication compounding is done in both inpatient and outpatient settings in the United States. Historically, compounded medications are prescribed by physicians when an FDA-approved medication is not available or cannot be used for a particular patient for medical reasons [4]. This should be done after the provider carefully analyzes the benefits-to-risk ratio.

These medications are especially valuable for patients who need a special dose or route of administration or when a patient is allergic to a particular ingredient in an FDA-approved medication [4]. Compounded medications may be helpful in improving patient adherence to medications, including topical medications [5]. This may be the case where a patient prefers a lotion instead of an ointment or in cases where the patient has better adherence with an orodispersible tablet than an oral conventional tablet [5].

In the pediatric population, extemporaneous compounding is done to prepare oral liquid formulations of medications in cases where the patient is not able to swallow oral solid capsules or tablets or if the required dosage cannot be provided with the oral solid medication [6]. Since the concentration of the compounded liquid medication can be different when prepared by different pharmacies, it can potentially lead to medication administration errors [6]. Efforts should be made to standardize the concentrations of liquid compounded medications for pediatric use to decrease the chances of errors [6].

Significant adverse events secondary to compounded medications

As pointed out by Watson et al., the risks of compounded medications broadly fall under two categories: those due to contamination and those due to inaccurate concentration or potency [2]. Poorly compounded medications that are contaminated can pose significant risks to patients due to lack of sterility. In 2012, the New England Compounding Center (NECC) shipped compounded sterile methylprednisolone acetate, which was used for epidural spinal injections and intra-articular injections. These steroid injections led to a meningitis outbreak leading to more than 700 infections and 64 deaths across the United States [2,7]. FDA investigation found environmental fungi in unopened vials of the methylprednisolone from NECC. It was found that the drug that was meant to be sterile was manufactured under inappropriate conditions [7]. Furthermore, while compounding should be used to manufacture custom-made medications for individual patients, various compounding pharmacies manufacture compounded medications on a large scale, thereby circumventing the guard rails put in place by the FDA to ensure drug quality, efficacy, and potency. FDA has received multiple reports of adverse reactions due to compounded medications before and after the 2012 meningitis outbreak. In 2017, 43 patients who had received an intravitreal injection of a compounded drug containing triamcinolone and moxifloxacin at the end of cataract surgery as a prophylactic measure against post-operative ocular inflammation and endophthalmitis reported a decrease in visual function and other visual symptoms [8]. Most recently, the GLP-1 agonists semaglutide and tirzepatide have been produced on a large scale by compounded pharmacies due to a shortage of these anti-obesity medications stemming from a supply-demand mismatch. Various adverse events have been reported to the FDA related to the compounded GLP-1 agonists due to incorrect dosing [9]. This may be due to patients self-administering incorrect doses or prescriptions of dosages higher than those approved by the FDA [9]. It has been reported that salt forms of semaglutide that have not been reviewed or approved by the FDA have also been used to manufacture compounded semaglutide [9]. Furthermore, prescribers may be held liable when an adverse event occurs due to a non-FDA-approved medication [4].

In the wake of the NECC incident, congress enacted the DQSA (Drug Quality and Security Act) [10]. Under this act, the FDA created a new category of “outsourcing facilities” [10]. These facilities voluntarily register with the FDA and are required to follow the GMP standards. These facilities are listed on the FDA website and are inspected by them according to a risk-based schedule [10]. The outsourcing facilities are allowed to do bulk compounding of medications and distribute medications across state lines [2].

How to maximize the safety of compounded medications

Healthcare providers should be aware of the distinction between FDA-approved and compounded medications, and the inherent risks of compounded medications. The compounded medications should only be used when an FDA-approved medication is not available. To ensure patient safety, pharmaceutical compounding must be done in the best possible conditions by certified practitioners (pharmacists), and validated standard operating procedures must be employed [11]. While manufacturing compounded medications, it is important to ensure that all ingredients have a certificate of analysis [11]. It is important to keep full records of the pharmaceutical production process [11]. The compounders must diligently calculate the quantity of each ingredient [2]. In the case of sterile preparations, staff must be adequately trained, and proper personal protective equipment must be used [2]. Furthermore, strict environment and air quality monitoring needs to be done, and disinfection must be maintained for compounding sterile preparations [2].

Lastly, it is important that when a provider decides to prescribe a compounded medication after carefully weighing the risks and benefits, the potential risks of the compounded product must be discussed with the patient, and the reason for using the compounded product must be documented [3].

Conclusion

Healthcare providers should be aware of the distinction between FDA-approved and compounded medications, and the inherent risks of compounded medications. FDA-approved medications undergo rigorous testing and consistently have higher manufacturing standards than compounded ones. Providers must use compounded medications only when an FDA-approved product is unavailable, the benefits are greater than the risks, and the potential risks have been discussed with the patient. There is a need for increased oversight of the compounding facilities and for supporting research into better regulatory frameworks and development of standardized safety measures for compounding pharmacies.
